# Woman with a Blackened Tongue: A Case Report

**DOI:** 10.5070/M5.52329

**Published:** 2026-04-30

**Authors:** Kyrillos Girgis, Cory Toomasian, Timothy Young

**Affiliations:** * Loma Linda University, Department of Emergency Medicine, Loma Linda, CA

## Abstract

Primary adrenal insufficiency is a rare disease characterized by deficient production of glucocorticoids with or without a deficiency of mineralocorticoids and adrenal androgens.^1^ This disease can present as a range of symptoms provoking a visit to the emergency department (ED). In this case, we present a 40-year-old female who reported tongue and gingival discoloration, unintentional weight loss, and skin hyperpigmentation. The patient was evaluated by an endocrinologist in the ED, received a dose of steroids in the ED, and discharged home with a prescription for steroids. This case report illustrates the distinctive tongue discoloration seen in primary adrenal insufficiency, highlighting the need for early recognition in the emergency setting to enable prompt and appropriate management.

**Topics:**

Endocrine, Addison’s disease, primary adrenal insufficiency, black tongue, steroids, skin hyperpigmentation.

**Figure:**
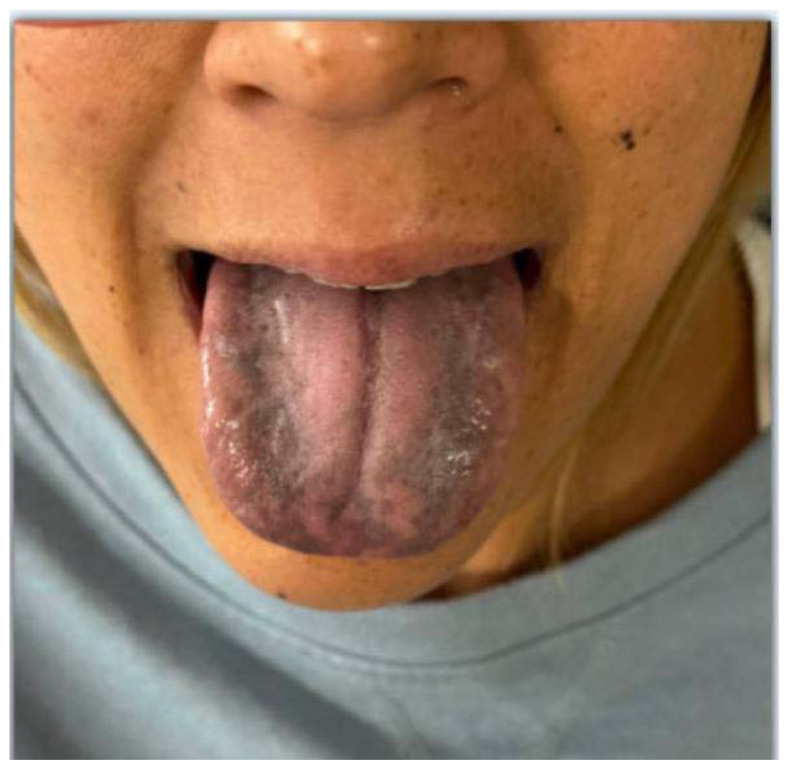


## Brief introduction

Primary adrenal insufficiency is a rare disease characterized by deficient production of glucocorticoids with or without a deficiency of mineralocorticoids and adrenal androgens.^1^ Clinical symptoms include weakness, fatigue, anorexia, abdominal pain, weight loss, orthostatic hypotension, and salt craving. 1 The cutaneous manifestations include hyperpigmentation of sun exposed areas, palmar creases, frictional surfaces, vermilion border, recent scars, genital skin, and oral mucosa.^2^ Cortisol deficiency leads to increased production of pro-opiomelanocortin, a prohormone cleaved into adrenocorticotropic hormone (ACTH) and melanocyte-stimulating hormone (MSH). Elevated MSH stimulates epidermal melanocytes, increasing melanin synthesis and causing hyperpigmentation.^3^ We present this case of characteristic tongue hyperpigmentation to raise awareness of Addison’s disease among emergency room clinicians. Written consent was obtained for publication of the image.

## Presenting concerns and clinical findings

A 40-year-old female with no past medical history presented to a California emergency department with tongue and gingival discoloration for the previous two months. The patient also complained of dizziness, a 33-pound unintentional weight loss, along with severe nausea and vomiting during the same time period. She did not have a history of using tanning beds or lotions. She was afebrile with otherwise normal vital signs. Her skin exam was notable for hyperpigmented skin with new developing nevi on her face.

## Significant findings

The patient’s vital signs were blood pressure 109/72 mmHg, respiratory rate 18 breaths per minute, pulse 93 beats per minute, temperature 97°F. Physical examination revealed notable black discoloration of the tongue and gingiva, along with hyperpigmented skin exhibiting numerous diffuse nevi distributed across the face and upper extremities.

## Patient course

The patient’s differential diagnosis in the ED included Addison’s disease, hypothyroidism, congenital adrenal hyperplasia, and hemochromatosis. The patient’s workup included a complete blood count, complete metabolic panel, thyroid panel, pregnancy test, adrenocorticotropic hormone (ACTH), cortisol level, and chest X-ray. Her workup revealed elevated ACTH, decreased cortisol levels, elevated thyroid stimulating hormone, normal potassium, hypochloremia, and hyponatremia. Her chest X-ray did not show any acute intrathoracic abnormalities. The endocrinologist evaluated the patient in the ED and recommended administering a steroid dose prior to discharge, followed by a prescription for outpatient use. The patient was scheduled for follow-up at the endocrinology clinic. Outpatient lab work revealed a positive 21-hydroxylase antibody, confirming the diagnosis of primary adrenal insufficiency. The patient was started on a mineralocorticoid and continues to follow up at the endocrinology clinic. She reports resolution of her dizziness and fatigue, though the tongue discoloration persists.

## Discussion

In early cases of Addison’s disease, symptoms are often subtle or nonspecific and can include fatigue, anorexia, weight loss, orthostatic hypotension, nausea, muscle and joint pain, or salt craving. Fortunately, our patient did not appear to be in adrenal crisis. Cutaneomucosal hyperpigmentation, seen in 92 percent of primary adrenal insufficiency cases, may appear as black, brown, bluish-black, or purple discoloration and can precede systemic manifestations.^4^ Occasionally, intraoral pigmentation might be the only sign of primary adrenal insufficiency. The buccal area is the most affected site; other sites are palatine arches, lips, gums, and tongue. Lingual pigmented patches may appear as isolated or multiple lesions on the dorsal and lateral surfaces. While typically asymptomatic, they can sometimes be associated with pain or itchiness.^4^ The cutaneous manifestations include darkening of the skin, especially in sun-exposed areas and hyperpigmentation of the palmar creases, frictional surfaces, vermilion border, recent scars, genital skin, and oral mucosa.^2^

In patients presenting with tongue discoloration and other nonspecific symptoms, a high index of suspicion is necessary to ensure timely diagnosis and treatment. Emergency physicians should initiate 100 mg intramuscular corticosteroid therapy without delay in suspected adrenal crisis because early intervention can significantly improve patient outcomes.^1^ Aside from steroid initiation, management of a patient with primary adrenal insufficiency in the ED focuses on prompt recognition and treatment of adrenal crisis, emphasizing fluid resuscitation and electrolyte correction.

Definitive diagnosis involves ACTH stimulation testing, in which 250 μg ACTH (cosyntropin) is given intravenously, and cortisol is measured at baseline, 30 minutes, and 60 minutes. A peak cortisol <18–20 μg/dL indicates adrenal insufficiency.^5^ If a clinician is highly suspicious of primary adrenal insufficiency, and the patient is stable for discharge home, a prescription for steroids should be given, with instructions to return to the ED if the patient develops fever, vomiting, diarrhea, significant blood loss, or shock.

As previously mentioned, when a patient presents to the emergency department with similar symptoms, it is important to consider alternative diagnoses in the differential, including but not limited to Addison’s disease, hypothyroidism, congenital adrenal hyperplasia, and hemochromatosis. Evaluation for hypothyroidism should include thyroid function tests, specifically thyroid-stimulating hormone (TSH) and free thyroxine (T4). Hemochromatosis can be assessed by obtaining iron studies, including serum ferritin and transferrin saturation, both of which are typically elevated.

This case highlights the importance of maintaining a high index of suspicion for primary adrenal insufficiency, particularly in patients presenting with subtle systemic symptoms and mucocutaneous hyperpigmentation, typically appearing as patchy or focal discoloration rather than diffuse involvement of the entire mucocutaneous region. While definitive diagnosis may not be achievable in the emergency department due to limited rapid testing options, early recognition and empiric corticosteroid therapy are critical to preventing adrenal crisis and reducing morbidity. As this case demonstrates, even rare and atypical presentations warrant vigilance, reinforcing the need for emergency physicians to be aware of this potentially life-threatening condition and to act decisively when it is suspected.

## Supplementary Information


