# Supplementary figures and images for: Woman with a Blackened Tongue: A Case Report
Source: J Educ Teach Emerg Med. 2026 Apr 30;11(2):V20–2. doi: 10.5070/M5.52329 (PMC13152370; doi:10.5070/M5.52329)

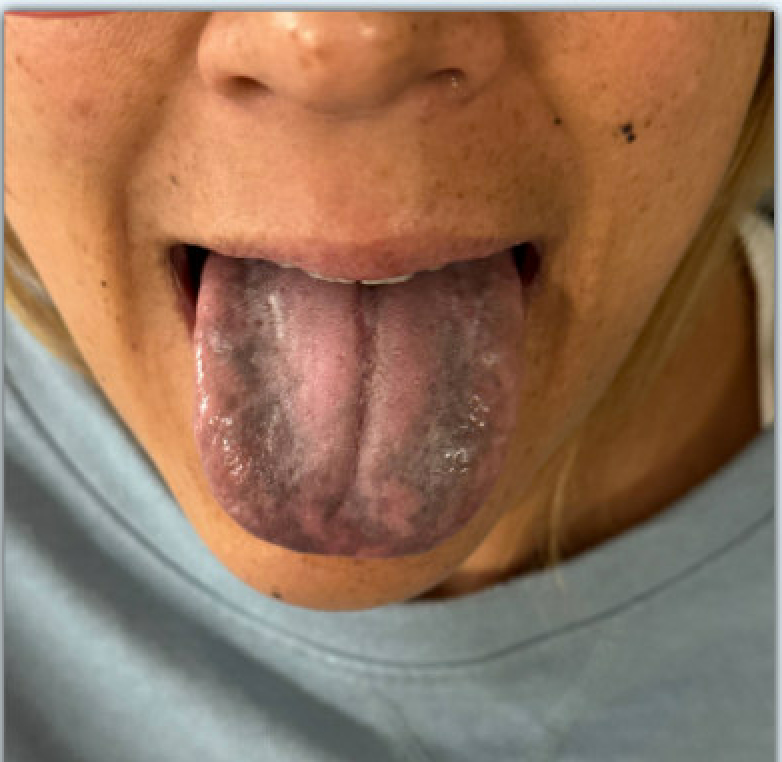

Supplement: Supplementary file 1 [file 11-2-V20-Supp1.jpg]
